# Effectiveness of rituximab in neuromyelitis optica: a meta-analysis

**DOI:** 10.1186/s12883-019-1261-2

**Published:** 2019-03-06

**Authors:** Fulin Gao, Bingyan Chai, Cheng Gu, Ruipeng Wu, Tong Dong, Yuping Yao, Yi Zhang

**Affiliations:** 1 Department of Neurology, Gansu Provincial Hospital, No. 204 of Donggang West Road, Lanzhou City, Gansu province 730000 People’s Republic of China; 2 School of Clinical Medicine, Gansu university of Traditional Chinese medicine, No. 35 of Dingxi East Road, Lanzhou City, Gansu province 730000 People’s Republic of China

**Keywords:** Neuromyelitis optica, Rituximab, Meta-analysis, Aquaporin-4 autoantibody, Annualized relapse rate, Expanded disability status scale

## Abstract

**Background:**

Neuromyelitis optica (NMO) is a severe inflammatory autoimmune disorder of the central nervous system and often results in paralysis or blindness. Rituximab (RTX) is a mouse–human chimeric monoclonal antibody specific for the CD20 antigen on B lymphocytes and used to treat many autoimmune diseases. Disability and relapses were measured using the Expanded Disability Status Scale (EDSS) and annualized relapse rate (ARR) ratio to evaluate the effectiveness of RTX. This review performed a meta-analysis of the efficacy of RTX in NMO.

**Methods:**

We searched through the databases of PubMed, Embase, and Cochrane Library. We compiled 26 studies, in which 18 used ARR ratio, 22 used EDSS score, and 14 used both variables. Differences in the ARR ratio and EDSS score before and after RTX therapy were used as the main efficacy measures. Publication bias was evaluated after the consistency test, and a sensitivity analysis was performed with mean difference (MD) of the efficacy of RTX.

**Results:**

A meta-analysis of 26 studies with 577 participants was conducted. Antibodies against aquaporin-4 autoantibody were recorded in 435 of 577 (75.39%) patients with NMO. RTX therapy resulted in a mean (WMD) − 1.56 (95% CI, − 1.82 to − 1.29) reduction in the mean ARR ratio and a mean (WMD) − 1.16 (95% CI, − 1.36 to − 0.96) reduction in the mean EDSS score. A total of 330 of 528 patients (62.9%) reached the relapse-free state. A total of 95 of 577 (16.46%) patients had adverse reactions.

**Conclusions:**

RTX has acceptable tolerance, reduces the relapse frequency, and improves disability in most patients with NMO. Future studies should focus on reducing the health-care costs, improving the functional outcomes, and reducing the adverse effects associated with RTX treatment.

## Background

Neuromyelitis optica (NMO) is a severe demyelinating disease that predominantly affects the optic nerve and spinal cord. The pathogenesis of NMO is related to aquaporin-4 autoantibody (AQP4-Ab) [1–3]. Serum antibodies targeting AQP4-Ab have become sensitive and specific biomarker for early diagnosis of NMO and are found in most patients. Prophylactic treatment of NMO recurrence must be immediately performed when NMO is identified because the progression of NMO disability is related to the severity of attacks. Considering that patients with NMO have antibodies against AQP4-Ab, several studies have proposed treatment for B cells in NMO [4].

Rituximab (RTX) is a chimeric monoclonal antibody directed against CD20 epitope expressed on pre-B and mature B cells and is used to treat B-cell-derived lymphoid neoplasms and antibody-mediated autoimmune diseases [5, 6]. The depletion of CD20 provides a theoretical basis for treatment of autoimmune diseases, in which B cells and autoantibodies play a key role; for example, AQP4-Ab is associated with NMO [7]. In this review, we performed a meta-analysis to evaluate RTX efficacy in terms of safety and tolerance and assessed the treatment efficacies based on relapse rates and disability.

## Methods

### Literature search

**Fig. 1:**
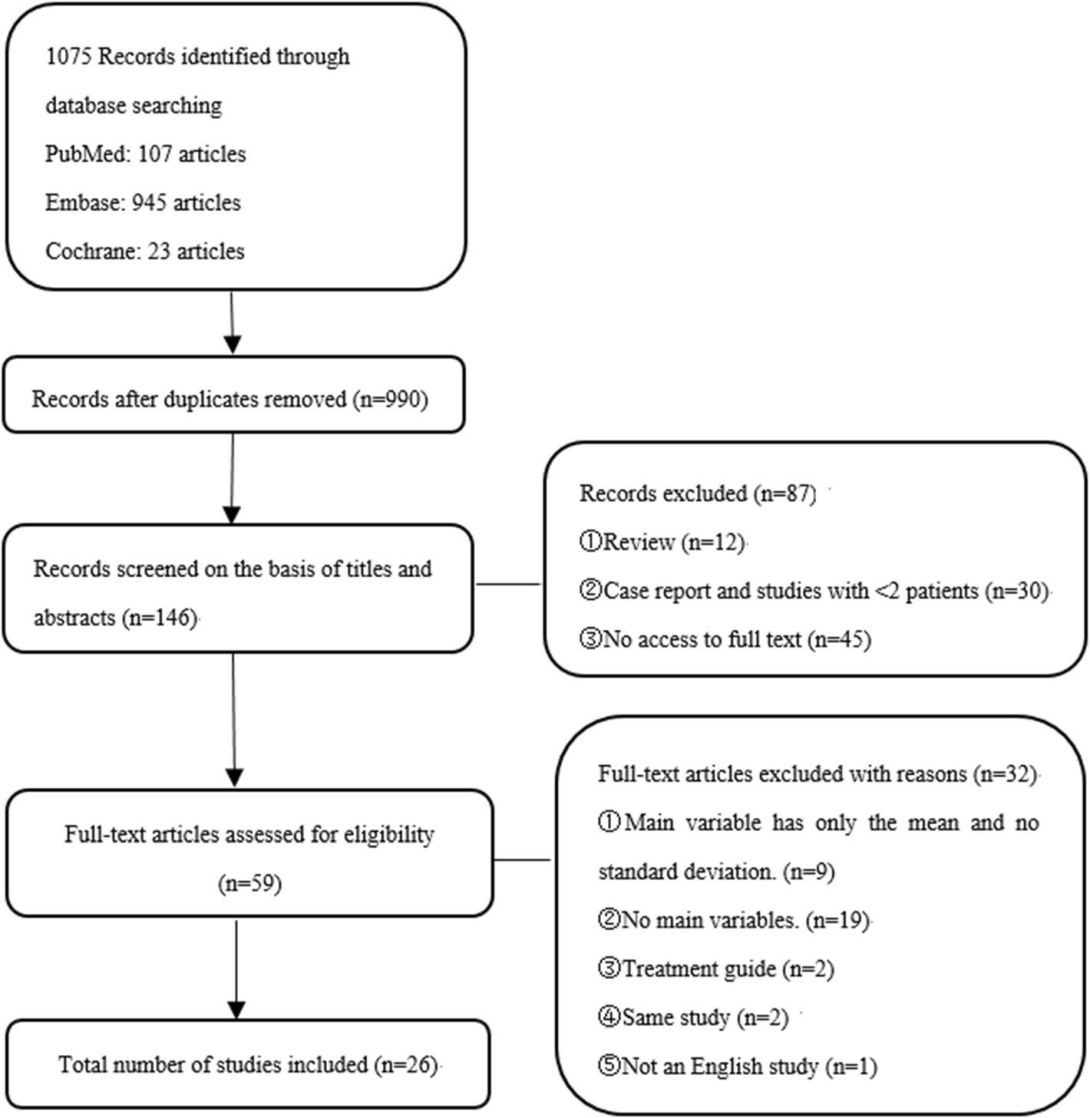
Flow chart presenting the process of the study selection for NMO meta-analysis

This search was restricted only to articles published in English language. We searched for publications on the PubMed, Embase, Cochrane Library, without any temporal restriction. We did keyword and Medical Subject Heading (MeSH) searches for our theme, and MeSH terms, key words and their synonyms related to “rituximab” and “neuromyelitis optica”. A flowchart of the search strategy is shown in Fig. 1. One of us used a standardized form of data extraction to extract data, another person checks it, and revisits the data that does not match, and resolves the differences through discussion and consensus.

### Inclusion and exclusion criteria for the literature

Studies were included if they fulfilled the following criteria: 1) Published English articles in various journals; 2) Patients with NMO do not limit their age, gender, ethnicity, and whether they have received treatment before; 3) Main variables include ARR and/or EDSS; Exclusion criterion: 1) Case reports and studies that included fewer than 2 patients, review, meta-analysis; 2) studies without main variables.

### Main variables

Among the 26 articles selected, we extracted the values (means and standard deviations) of EDSS and ARR directly available. Disability was measured by the EDSS. The ARR were calculated using the total number of relapses per patient-year.

### Statistical analysis

Data analysis was performed using statistical software provided by State 12.0. The heterogeneity across each effect size was evaluated with the I^2^ index, in which I^2^ value close to 0% indicates no heterogeneity between studies, close to 25% indicates low heterogeneity, close to 50% indicates moderate heterogeneity and close to 75% indicates high heterogeneity between studies. If *P* > 0.1, I^2^ ≤ 50%, a fixed-effect model was used for meta-analysis. When *P* < 0.1, I^2^ > 50%, a random-effect model was used instead and meta-regression analyzed the causes of heterogeneity, such as age of onset, duration of disease, follow-up time, dose of infusion and AQP4-IgG serostatus. A *P* value < 0.05 was considered as clinical significance.

## Results

### Study identification and selection

By searching PubMed, Embase, and Cochrane library database dated until August 2018. The database search identified 1075 records. After removing duplicates, 990 titles were initially screened and 146 theme-related abstracts were selected for further screening. Finally, 26 studies were included in this systematic review. 18 used ARR ratio, 22 used EDSS score, and there are 14 studies in the two main variables.

### Demographic and clinical characteristics

Table 1 lists detailed information from 26 included studies. The included studies were published between 2008 and 2018. The number of participants per study ranged from 3 to 100, with a total number of 577(503 females and 67 males, with sex not specified in 7 patients). NMO patients have antibodies against AQP4-Ab were recorded in 435 of 577 (75.39%).

**Table 1:** Clinical and demographic characteristics of 577 patients from 26 studies included in the systematic review

Reference (study)	Research type	Patient No.	Sex (F/M)	Age (year)	AQP4-Ab (+) (case)	Duration of disease (years/month)	Follow-up (years/month)
Jacob [11] 2008	Retrospective	25	22/3	38(7–65)	11	4.5(0.8–17)y	19(6–40)m
Jarius [14] 2008	Retrospective	4	NC	45(19–59)	4	NC	62(33–144)m
Pellkofer [16] 2011	Prospective	9	8/1	36.1(11.5)	9	11(7.7)y	29.6(14.5)m
Gurdesh [2] 2011	Retrospective	23	21/2	37.1(14.6)	15	114(13–266)m	32.5(7–63)m
Lindsey [26] 2012	Retrospective	8	7/1	37.6(14.4)	4	65.1(53.7)m	39.9(40.7)m
Yang [20] 2013	Retrospective	3	NC	34.3(8.5)	2	9.3(4)y	12.7(0.6)m
Ip [5] 2013	Retrospective	7	6/1	52(22–62)	4	57(40–272)m	24(1–42)m
Ayzenberg [27] 2013	Retrospective	3	3/0	35(7.8)	3	6.7(3.7)y	14.7(15.1)m
Gredler [28] 2013	observational	4	4/0	42.5(15.4)	4	6.2(4.2)y	3.1(2.1)y
Chay [10] 2013	Retrospective	6	4/2	37.8(20.6)	3	NC	NC
Longoni [25] 2014	Retrospective	5	4/1	13.7(2.7)	5	3.2(0.3)y	21.5(6.9)m
Kim [23] 2015	Retrospective	100	92/8	43(11)	94	11(5)y	67(9–108)m
Zephir [22] 2015	Retrospective	32	27/5	45(12.1)	28	6.5(1–410)m	28.7(21)m
Weinfurtner [18] 2015	Retrospective	4	3/1	26.5(22.3)	3	6.5(3.1)y	6(1.2)y
Jeong [29] 2015	Retrospective	55	50/5	42(15–68)	52	41.7(2.1–231.5)m	64.7(6.2–99.8)m
Valentino [7] 2016	Retrospective	7	6/1	38.3(16.6)	7	NC	59.4(29.7)m
Annovazzi [1] 2016	Retrospective	76	64/9	46.5(12.5)	53	6(7.2)y	35.6(27)m
Collongues [30] 2016	Retrospective	21	19/2	37.8(15.5)	19	46.9(51.2)m	31(18)m
Zhang [31] 2017	Case-control	31	23/8	42.2(16.9)	15	4.05(2.11)y	27.45(11.68)m
Nikoo [32]2017	RCT	33	29/4	35.33(8.98)	13	6.23(4.29)y	> 12 m
Evange. [17]2017	Retrospective	5	5/0	54(10.21)	5	6.8(1.3)y	6.6(0.9)y
Cohen [33] 2017	Prospective	40	33/7	40.2(22–62)	20	40(2–165)m	2y
Tallantyre [34] 2018	Retrospective	5	5/0	36.6(14.5)	5	11.5(9.4)y	3.5(0.2–8.9)y
Yang [15] 2018	Prospective	20	19/1	40.7(11.4)	10	11(0.2–240)m	29(18–40)m
Cabre [35] 2018	Prospective	32	30/2	39.9(12.1)	20	NC	2y
Li [21] 2018	Retrospective	19	16/3	34.8(13.7)	17	3.4(3.4)y	2.5(1.7)y

*RCT* Randomized clinical trial, *AQP4-Ab* Aquaporin 4 autoantibody, *NC* No clear

### Efficacy on the ARR ratio

**Fig. 2:**
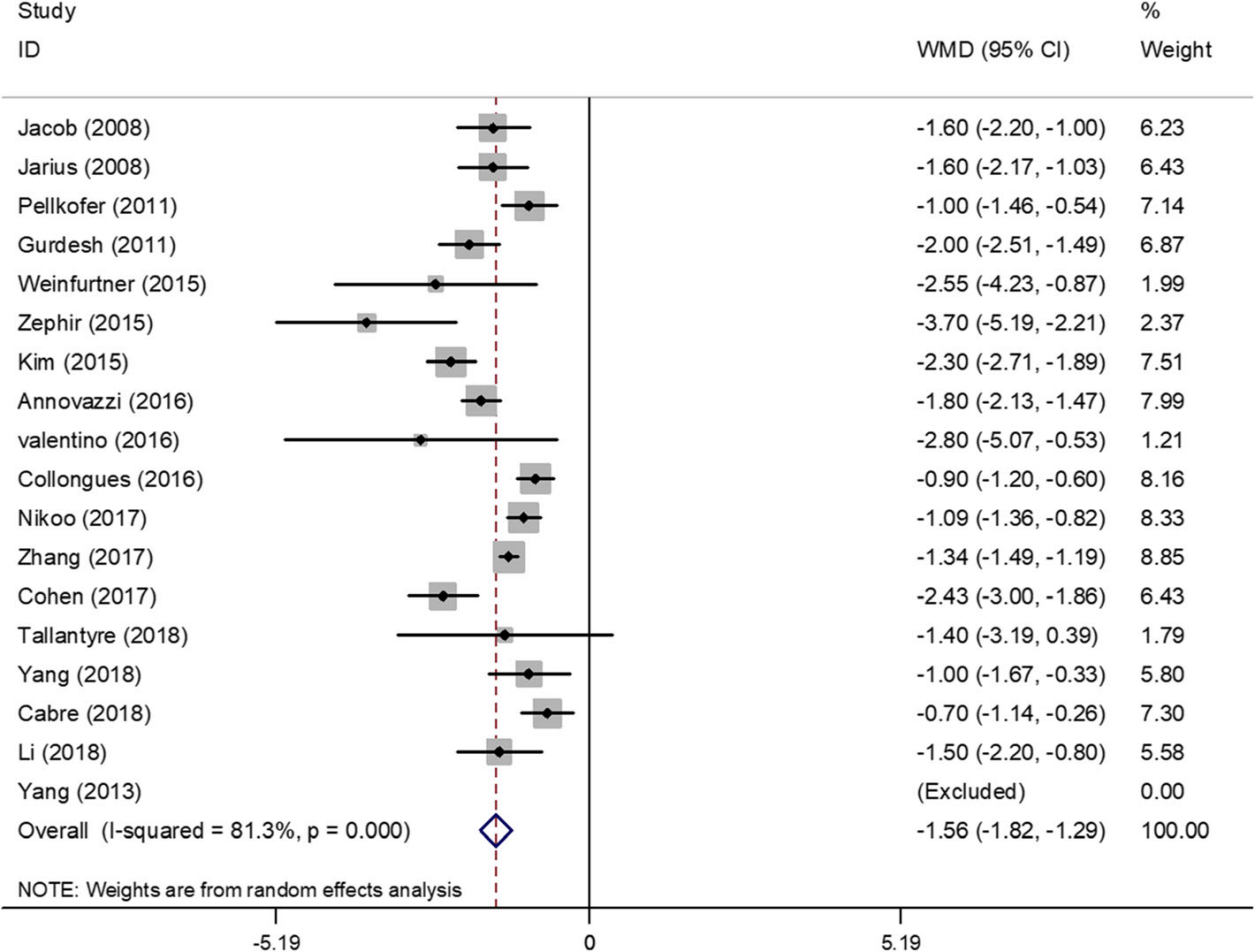
Forest plot of the mean difference in the ARR ratio before and after rituximab therapy. The three patients of Yang^2013^ had no relapse after treatment and could not be estimated in the forest plot. The estimated pooled weighted mean difference was −1.56 was highly significant (*p* < 0.0001), however, there was a large heterogeneity of study results (I2 = 81.3%)

Figure 2 shows a forest plot of the mean difference in the ARR ratio before and after rituximab therapy. This finding suggested that the mean difference of ARR ratio after rituximab therapy was − 1.56 (95%CI, − 1.82 to − 1.29). A random-effect model was used with I^2^ of 81.3%. Sensitivity analyses were performed by removing each study in turn and re-analyzed. No studies found to significantly affect heterogeneity. To evaluate the effect of the different covariates on the ARR ratio reduction, a meta-regression was performed. No significant correlation was detected between the outcome (ARR ratio change) and the following variables: age of onset (*P* = 0.80; 95% CI, − 0.29 to 0.23), duration of disease (*P* = 0.77; 95% CI, − 0.02 to 0.02), follow-up time (*P* = 0.90; 95% CI, − 0.07 to 0.06), dose of infusion (*P* = 0.77; 95% CI, − 0.52 to 0.67) and AQP4-IgG serostatus (*P* = 0.78; 95% CI, − 3.00 to 3.81).

### Efficacy on the EDSS score

**Fig. 3:**
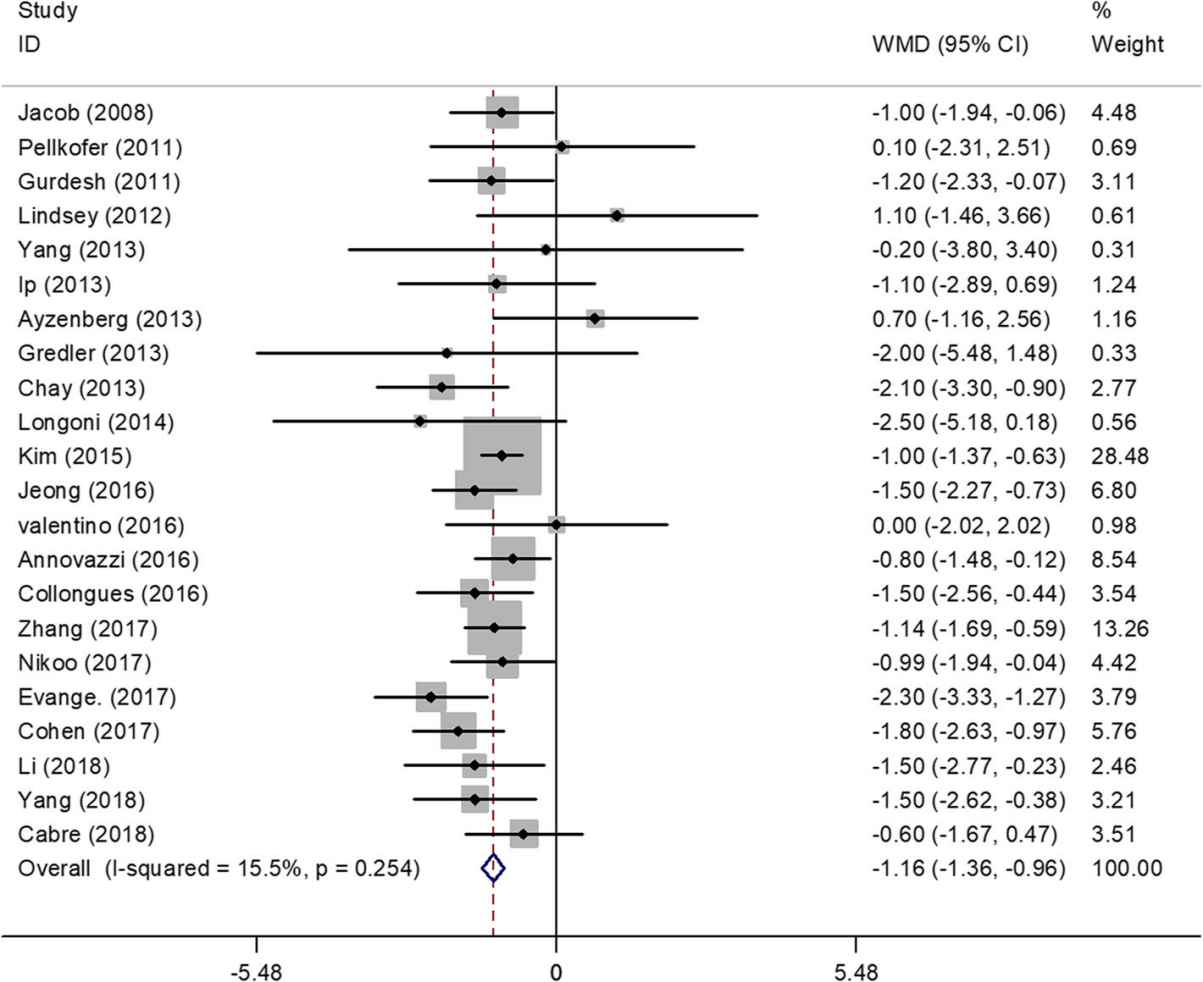
Forest Plot Showing the EDSS score of Patients with NMO after Rituximab Therapy. The estimated pooled weighted mean difference was − 1.16 was highly significant (*p* < 0.0001), there was a moderate heterogeneity of study results (I2 = 15.5%)

Figure 3 shows a forest plot of the mean difference in the EDSS score before and after rituximab therapy. This finding suggested that the mean difference of EDSS score after rituximab therapy was − 1.16 (95%CI, − 1.36 to − 0.96). The heterogeneity was moderate (I^2^ = 15.5%, *P* = 0.254). No significant correlation was detected between the outcome (EDSS Score change) and the following variables: age of onset (*P* = 0.48; 95% CI, − 0.08 to 0.42), duration of disease (*P* = 0.70; 95% CI, − 0.01 to 0.01), follow-up time (*P* = 0.23; 95% CI, − 0.01 to 0.02), dose of infusion (*P* = 0.88; 95% CI, − 0.24 to 0.21) and AQP4-IgG serostatus (P = 0.23; 95% CI, − 2.66 to 0.70).

### Safety

330 of 528 patients (62.9%) reached a relapse-free state. Adverse effects were recorded in 95 of 577 (16.46%) patients treated with rituximab. Twelve of the patients experienced severe adverse reactions, five patients developed severe pneumonia, two patients developed transit hyperpyrexia, two patients developed septicemia, one patient developed a severe allergic reaction, one patient had a urogenital infection, and one patient developed seborrheic dermatitis. Five patients died. Two patients died of pneumonia, one patient died of urogenital infection and thrombosis, one patient died of bone marrow transplantation, and one patient died of cardiac and respiratory failure due to very extensive myelitis reaching the medulla oblongata.

### Publication bias

**Fig. 4:**
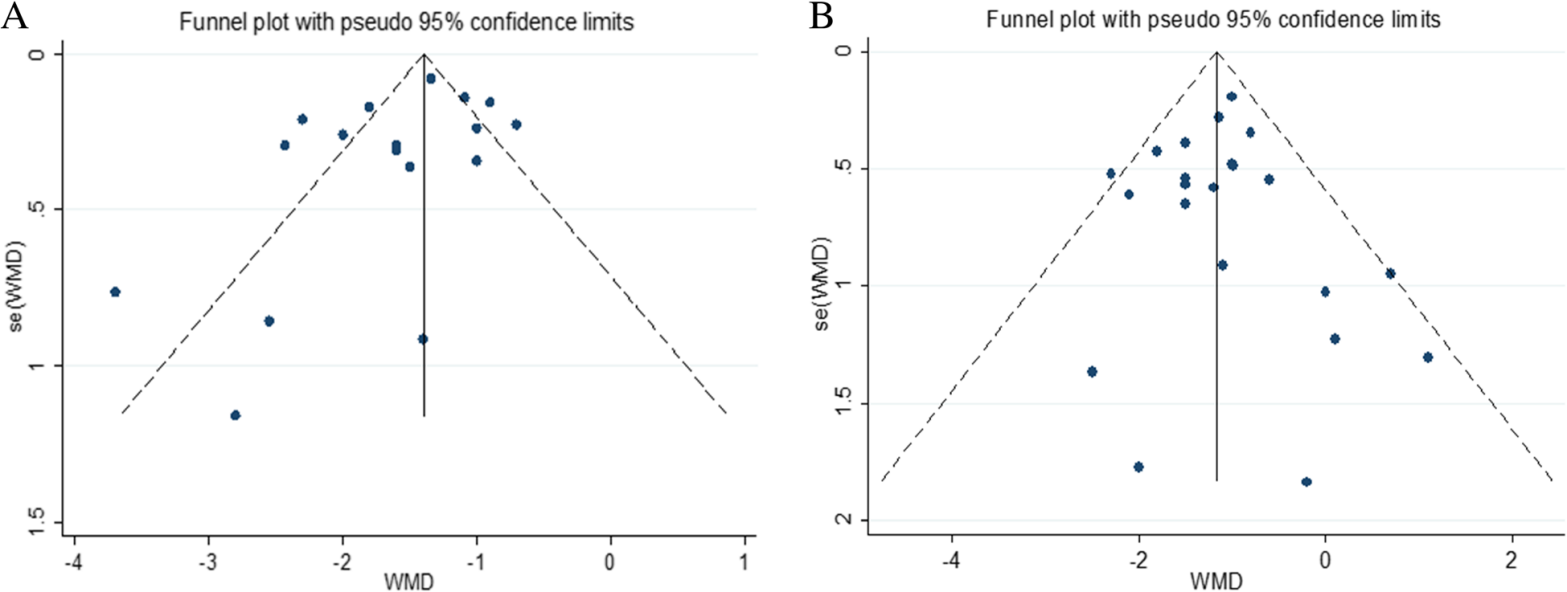
Funnel plot showing the incidence of ARR and EDSS of Patients with NMO after Rituximab Therapy. The funnel plot for studies on the incidence of ARR were generally symmetrical (*P* = 0.135;95% CI, −3.53 to 0.52) (**a**). The funnel plot for studies on the incidence of EDSS were symmetrical (*P* = 0.792; 95% CI, −0.84 to 1.09) (**b**)

The funnel plot for studies on the incidence of ARR and EDSS were symmetrical. The funnel plots indicated an absence of publication bias. (Fig. 4).

## Discussion

NMO is a relapsing disease with a high early mortality rate. More than 50% of patients with NMO will be functionally blind or will progress to wheelchair dependence within 5 years without employing appropriate immunosuppressant treatment [8, 9]. Treatment options for NMO are based on case series and expert opinion; among which, immunosuppressive therapy is the main method used to prevent recurrence and disability. Successful use of RTX has been widely reported in NMO. However, randomized controlled trials in NMO are relatively few, and no established guidelines have been established for RTX treatment. Although RTX is expensive, it can offset the cost of recurrence and plasma exchange due to its good therapeutic effect [10, 11]. At present, RTX has been biosimilarized, and its price has been gradually accepted by patients.

The therapeutic effect of RTX varies among patients. We performed a meta-regression and analyzed the causes of heterogeneity. We found no significant correlation among the age of onset, duration of disease, follow-up time, dose of infusion, AQP4-IgG serostatus, and major variables (ARR and EDSS). We speculated that the causes of heterogeneity in previous studies may be related to differences in ethnicity, study design, and inclusion criteria and whether other treatments are received prior to treatment. The selection of the right therapeutic agent for a patient is important due to the increasing number of treatment options. RTX is used as a prophylactic first-line treatment because it can reduce the severity of disease recurrence [12, 13].

Although AQP4-Ab is critical in the diagnosis of NMO, its involvement in the pathogenesis of the disease remains controversial [9]. Several studies have suggested that AQP4-Ab is generally used as a marker of disease activity in an individual patient [14, 15]. However, many studies have shown that AQP4-Ab is only a diagnostic marker for NMO and can only be detected in serum during relapse and remission [16, 17]. Patients with partial AQP4-Ab negative NMO still have a good response to RTX treatment [18]. Maintaining the consumption of memory B cells through repeated treatment may be pivotal to the clinical effects of RTX in patients with NMO [19]. CD27+ memory B cells can better detect the therapeutic effect of RTX than CD19+ B cells.

Yang et al. [20] found that low RTX doses administered to Asian patients with NMO can achieve high reactivity and have cost and availability advantages. Low-dose RTX can effectively reduce the recurrence rate and improve the prognosis in most NMO cases. Some patients require an increased frequency of RTX infusion to maintain low levels of CD19+ cells, and long-term use of low-dose RTX may lead to cost reduction [17, 21]. Infections are the commonly reported adverse drug reactions. In some patients, the immunoglobulin levels decreased following RTX treatment, thereby increasing their risk of infection [22, 23]. Serious adverse reactions leading to death were rarely reported. In our meta-analysis, five patients died due to serious illness and related complications. Only two of these deaths were associated with adverse reactions to RTX. One patient died from pneumonia, and the other patient died from urogenital infection and thrombosis.

Our meta-analysis aimed to present efficacy data and to expand knowledge about the safety of RTX treatment. Although RTX has important benefits for treatment of NMO, its long-term benefits and risks remain to be determined. Moreover, most patients receive other immunotherapies before and after RTX treatment so the benefits and risks of treatment using a single drug are inaccurate [5, 18, 24, 25]. And it is unclear whether patients have the appropriate time to discontinue RTX treatment without the risk of further relapse. In the previous meta-analysis, there was no mention of publication bias. It was also found that the duration of the disease and the efficacy measures showed a significant correlation. However, we did not find a correlation between disease duration and efficacy measures in our meta-analysis. This may be related to the differences we have included. Limitations of this study:1) Although the search strategy is relatively complete, it does not rule out that eligible articles are not included. 2) A large sample of multicenter studies was lacking in the included studies.

## Conclusions

RTX has acceptable tolerance, reduces the relapse frequency, and improves disability in most patients with NMO. Future studies should focus on reducing the health-care costs, improving the functional outcomes, and reducing the adverse effects associated with RTX treatment.

## Abbreviations

AQP4-Ab: aquaporin-4 autoantibody
ARR: annualized relapse rate
AZA: acetazolamide
EDSS: Expanded Disability Status Scale
MD: mean difference
MeSH: Medical Subject Heading
NMO: Neuromyelitis optica
RCT: randomized clinical trial
RTX: Rituximab
